# LIK1, A CERK1-Interacting Kinase, Regulates Plant Immune Responses in *Arabidopsis*

**DOI:** 10.1371/journal.pone.0102245

**Published:** 2014-07-18

**Authors:** Mi Ha Le, Yangrong Cao, Xue-Cheng Zhang, Gary Stacey

**Affiliations:** Divisions of Plant Sciences and Biochemistry, National Center for Soybean Biotechnology, C.S. Bond Life Sciences Center, University of Missouri, Columbia, Missouri, United States of America; University of Wisconsin-Madison, United States of America

## Abstract

Chitin, an integral component of the fungal cell wall, is one of the best-studied microbe-associated molecular patterns. Previous work identified a LysM receptor-like kinase (LysM-RLK1/CERK1) as the primary chitin receptor in *Arabidopsis*. In order to identify proteins that interact with CERK1, we conducted a yeast two-hybrid screen using the intracellular kinase domain of CERK1 as the bait. This screen identified 54 putative CERK1-interactors. Screening mutants defective in 43 of these interacting proteins identified only two, a calmodulin like protein (At3g10190) and a leucine-rich repeat receptor like kinase (At3g14840), which differed in their response to pathogen challenge. In the present work, we focused on characterizing the LRR-RLK gene where mutations altered responses to chitin elicitation. This LRR-RLK was named LysM RLK1-interacting kinase 1 (LIK1). The interaction between CERK1 and LIK1 was confirmed by co-immunoprecipitation using protoplasts and transgenic plants. *In vitro* experiments showed that LIK1 was directly phosphorylated by CERK1. *In vivo* phosphorylation assays showed that Col-0 wild-type plants have more phosphorylated LIK1 than *cerk1* mutant plants, suggesting that LIK1 may be directly phosphorylated by CERK1. *Lik1* mutant plants showed an enhanced response to both chitin and flagellin elicitors. In comparison to the wild-type plants, *lik1* mutant plants were more resistant to the hemibiotrophic pathogen *Pseudomonas syringae*, but more susceptible to the necrotrophic pathogen *Sclerotinia sclerotiorum*. Consistent with the enhanced susceptibility to necrotrophs, *lik1* mutants showed reduced expression of genes involved in jasmonic acid and ethylene signaling pathways. These data suggest that LIK1 directly interacts with CERK1 and regulates MAMP-triggered innate immunity.

## Introduction

Chitin, a polymer of β-1,4 linked N-acetyl glucosamine, is an important component of the fungal cell wall. Among the best-studied microbe-associated molecular patterns (MAMPs), it is capable of eliciting basal defense responses in plants against fungal pathogens. Receptors for chitin include the chitin elicitor binding protein (OsCEBiP) in rice [1] and LysM-containing receptor-like kinase LysM RLK1/CERK1 (chitin elicitor receptor kinase) in *Arabidopsis* [2], [3]. All known plant chitin receptors contain extracellular LysM domains, which are ancient, ubiquitous protein modules capable of binding peptidoglycan and structurally-related molecules [4], [5]. *Arabidopsis* CERK1 contains three extracellular LysM motifs, a transmembrane domain, and an intracellular kinase domain [2]; whereas OsCEBiP has two extracellular LysM motifs but lacks an intracellular domain [1], [3]. In addition to OsCEBiP, chitin signaling in rice was shown to require OsCERK1, the ortholog of AtCERK1 [6]. Using a pair wise yeast two-hybrid method, the extracellular domains of OsCEBiP and OsCERK1 were shown to interact, and the extracellular domain of each protein was shown to form a homodimer [6]. Interestingly, in *Arabidopsis*, another LysM receptor like kinase, LYK4, was also shown to be involved in chitin-induced innate immunity [7]. AtLYK4 has an inactive kinase, based on the lack of key amino acid residues in the kinase domain, as well as the lack of *in vitro* kinase activity [7]. *Arabidopsis* LYM1, LYM2, and LYM3, all with similar structure to OsCEBiP, are not involved in chitin-triggered innate immune responses determined by ROS production and downstream gene expression [7], [8]. However, chitin-induced molecular flux *via* plasmodesmata was inhibited in *lym2* mutant plants but not in *lym1* or *lym3* mutant plants [9]. In addition, *lym2* mutant plants were more susceptible to fungal pathogens *Botrytis cinerea* and *Alternaria brassicicola* [9], [10]. Therefore, *Arabidopsis*, unlike rice in which OsCERK1 and OsCEBiP likely form a heterodimeric receptor complex, differs in not using the OsCEBiP orthologs but perhaps a receptor composed of AtCERK1 and AtLYK4, as well as other uncharacterized proteins [11]. However, rice and *Arabidopsis* may be similar in that the receptor complex is composed of one transmembrane receptor protein possessing active kinase activity (i.e., CERK1) and a co-receptor lacking an active intracellular kinase domain (i.e., either OsCEBiP or AtLYK4).

Although originally identified as a chitin receptor, recently AtCERK1 was also shown to be required for the plant response to bacterial cell wall-derived peptidoglycan (PGN) [12]. The data suggest that AtCERK1 interacts with the glycosylphosphatidylinositol-anchored LysM proteins, AtLYM1 and AtLYM3, to form the PGN receptor complex. Consistent with the model proposed for OsCERK1-OsCEBiP, it was hypothesized that AtLYM1 and AtLYM3, both lacking a kinase domain, may bind to PGN, which activates intracellular signaling pathways via activation of AtCERK1 kinase activity [12]. However, although this model is supported by mutant analysis, there is no direct biochemical evidence that AtLYM1-LYM3 and AtCERK1 directly interact.

The critical role of AtCERK1 in chitin perception has been confirmed by a variety of molecular, genetic [2], [3], and biochemical studies [13], [14]. For example, *AtCERK1* mutant plants are impaired in all chitin responses, including reactive oxygen species (ROS) production, the activation of a MAPK cascade, and the expression of chitin-induced genes, eventually resulting in the failure of chitin-induced pathogen resistance [2], [3]. Biochemical analysis confirmed direct binding between AtCERK1 and chitin [13], [14], albeit at a much lower affinity (µM) than predicted by physiological assays that measured the response of plants to chitin elicitation (<nM). Recently, the X-ray crystal structure of the extracellular LysM domain of AtCERK1 (AtCERK1-ECD) was elucidated [15]. The structure predicts that a single AtCERK1 monomer can bind chitotetraose, but such binding results in little or no induction of MAMP- triggered immunity (MTI). Binding of longer chitooligomers (d.p.>7) resulted in homodimerization of AtCERK1. Since these longer chain chitooligomers are required to induce a strong MTI response [16], the authors suggested that homodimerization of AtCERK1 may be essential for signal transduction [15].

Other proteins are also known to modulate chitin signaling via direct interaction with AtCERK1. For example, AvrPtoB, a type III secretion system effector from *Pseudomonas syringae* with ubiquitin E3 ligase activity, interacts with the kinase domain of AtCERK1 and inhibits its kinase activity, presumably by promoting the ubiquitination of the receptor [17], [18]. AtCERK1 also appears to function in conjunction with a variety of putative co-receptors or co-adaptors. For example, AtCERK1 was found to interact with BIK1 [19]. Interestingly, BIK1 also interacts with FLS2, the cognate receptor for flagellin (e.g., the flg22 peptide), a strong elicitor of innate immunity in *Arabidopsis* [19], [20]. BIK1 is required for the phosphorylation activity of the FLS2/BAK1 receptor complex, as well as for flg22-triggered immunity [19], [20]. Mutant plants defective in BIK1 showed a reduction in chitin responses, including ROS production and callose deposition [19]. However, it is not clear if the interaction between BIK1 and AtCERK1 is required for chitin signaling or for chitin-triggered immunity.

In order to identify novel components that directly interact with AtCERK1, a yeast two-hybrid screen was used to identify 54 putative AtCERK1 interactors of which we were able to identify mutant lines in 43 genes. We initially screened these 43 lines by monitoring the accumulation of ROS in response to chitin elicitation and then subsequently for their pathogen response. This resulted in the identification of only two mutant lines that showed a significantly altered response to pathogen inoculation; that is mutations in At3g10190, encoding a calmodulin-like protein, and At3g14840, encoding a LRR-RLK. Since mutants lacking the calmodulin-like protein retained the ability to respond to chitin, albeit at a significantly lower level than the wild-type, we focused our attention on the LRR-RLK protein, LIK1, where mutations enhanced the response to chitin elicitaion. The interaction between AtCERK1 and LIK1 was confirmed by co-immunoprecipitation. An *in vitro* kinase assay showed that LIK1 has very low, but measurable kinase activity. Wild-type LIK1 protein, as well as a mutant form lacking kinase activity, were phosphorylated *in vitro* upon the addition of CERK1. Interestingly, *lik1* mutant plants showed increased resistance to the hemibiotrophic pathogen, *P.syringae* pv. *tomato* DC3000, but increased susceptibility to the necrotrophic fungal pathogen, *S. sclerotiorum*. Collectively, these data suggest that LIK1 is part of the AtCERK1 receptor complex and negatively regulates chitin-induced immunity.

## Results

### Identification of the CERK1 interacting kinase (LIK1)

To identify new components interacting with AtCERK1, we performed a yeast two-hybrid screen using a cDNA library produced from chitin-treated seedlings (screening procedure described in Figure S1). This screen identified 54 putative AtCERK1 interactors (Table S1). T-DNA insertion mutants were acquired for the majority (43 out of 54) of these interactors and the mutants were subsequently screened for their ability to produce ROS upon chitin elicitation. This secondary screen led to the identification of 16 mutant lines that produced either more or less ROS upon chitin elicitation (Figure S2). These 16 mutant lines were subsequently challenged with the bacterial pathogen, *P. syringae* pv. *tomato* DC3000, leading to the identification of two mutants with an altered pathogen response (Figure S3). Compared with the wild-type, the calmodulin-like protein mutant plants showed lower responses to chitin elicitation in both the ROS production assay (Figure S2) and the bacterial pathogen assay (Figure S3). *Lik1* mutant plants showed an enhanced response to chitin in the ROS production assay (Figure S2), as well as an increased resistance to *P. syringae* pv. *tomato* (Figure S3). In this study, we focused on further characterization of LIK1. The *LIK1* gene spans 5952 bp and consists of 23 introns and 24 exons. The gene model and predicted protein structure suggest that the gene encodes a protein of 1,021 amino acids that contains an extracellular LRR domain, a transmembrane domain and an intracellular Ser/Thr kinase domain (Figure S4A and S4B). A total of four unique T-DNA insertion lines were obtained for AT3G14840 and named *lik1-1, lik1-2, lik1-3* and *lik1-4* with the insertions located in intron 2, intron 13, exon 18 and exon 18, respectively. These four mutants were further confirmed using reverse transcript PCR to amplify the 3'-end of the gene. As shown in Figure S4C, no PCR product was amplified from these four mutants compared with the Col-0 wild-type *Arabidopsis*, confirming that transcription of the gene is blocked in all four mutants.

### LIK1 interacts with CERK1 *in vivo*

**Figure 1:**
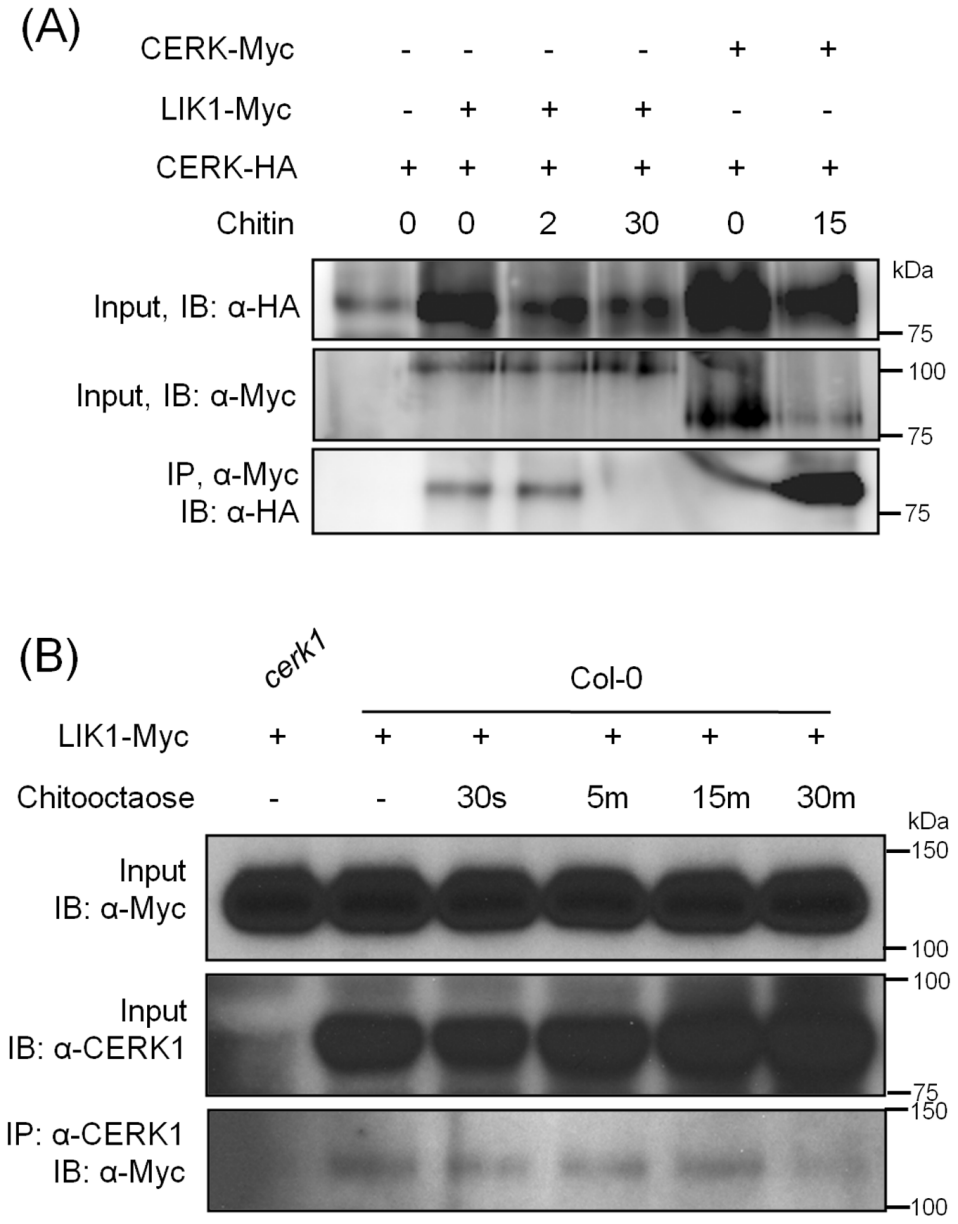
AtLIK1 interacts with AtCERK1. (A) HA-tagged full-length AtCERK1 and Myc-tagged full length AtLIK1 or AtCERK1 were co-expressed under the control of the CaMV35S promoter in *Arabidopsis* Col-0 protoplasts. Protoplasts were harvested prior to (-) or at different time points (as shown in figure) after treatment with 100 µg/ml chitin. Co-immunoprecipitation was carried out using anti-cMyc antibody. Input blots are from SDS-PAGE of total protein extracts; each lane was loaded with an equivalent volume of total protoplasts. IB: antibody used to probe immunoblot. (B) Myc-tagged *LIK1* was transgenically expressed in Col-0 wild-type and *cerk1* mutant plants. 10-day-old transgenic seedlings were treated with chitooctase for the time point as shown in the figure. Co-immunoprecipitation was made using anti-CERK1 antibody and detected with anti-Myc antibody.

Since the Y2H methods often generate many false positive results, especially given that our screen was saturated, exceeding 4.5×10^6^ transformants, we sought to confirm the LIK1-CERK1 interaction using co-immunoprecipitaion. A LIK1 fusion with a 4×Myc tag and a CERK1 fusion with a 3×HA tag, both expressed from the strong CaMV35S promoter, were transiently co-expressed in *Arabidopsis* protoplasts. Proteins extracted from these protoplasts were then precipitated using anti-cMyc antibody. As shown in Figure 1A, LIK1 can immunoprecipitate CERK1 before and after treatment with chitooctaose. However, the interaction between CERK1 and LIK1 decreased 30 minutes after chitin treatment.

To further confirm the interaction between CERK1 and LIK1, *LIK1* fused with 4×Myc tag was transformed into Col-0 and *cerk1* mutant plants under the control of *35S* CaMV promoter. LIK1 protein expression was detected using anti-Myc antibody (Figure S6). Multiple T2 transgenic seedlings were used for co-immunoprecipitation assay using anti-CERK1 antibody. As shown in Figure 1B and Figure S5, LIK1 was co-immunoprecipitated with CERK1 before and after chitooctaose treatment. The interaction between CERK1 and LIK1 was slightly reduced at 30 min after treatment with chitooctaose.

### CERK1 phosphorylates LIK1 *in vitro*

**Figure 2:**
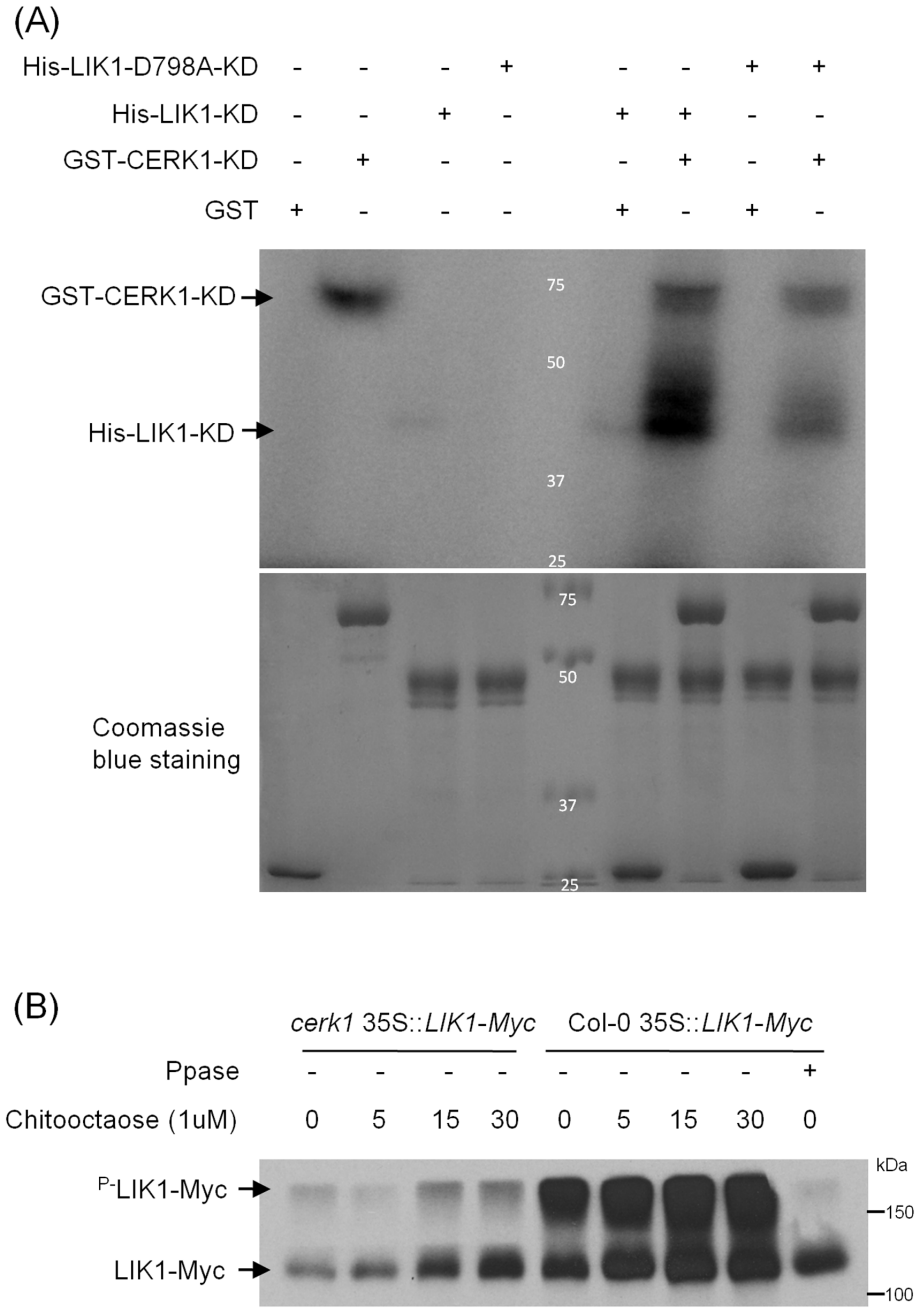
AtCERK1 phosphorylates AtLIK1. (A) GST fusion AtCERK1 kinase domain and His-tag fusion AtLIK1and AtLIK1^D798A^ kinase domain were purified from *E. coli* for the *in vitro* kinase assay. About 1 µg protein was used for the kinase assay in the presence of [γ-^32^P]-ATP. Upper panel is the autoradiography and lower panel is the Coomassie blue stained image. (B) LIK1 phosphorylation in response to chitooctaose treatment in *cerk1* mutant and Col-0 wild-type plants. Samples were separated on phos-tag SDS-PAGE gel to reveal phosphorylation of LIK1 and then subjected to western blot using anti-Myc antibody.

The fact that CERK1 co-immnoprecipitates with LIK1 and both are predicted to be Ser/Thr kinases prompted us to explore the possibility of cross-phosphorylation. We performed *in vitro* phosphorylation assays using the CERK1 kinase domain (254 a.a. to 617a.a.) fused with the glutathione sulfotransferase domain and LIK1 kinase domain (644 a.a. to 1, 020 a.a.) fused with a 6 × His motif. These two proteins were expressed in *E. coli* and purified by affinity chromatography. We also made an inactive kinase derivative of LIK1 (D798A) by mutating an aspartate residue in the kinase catalytic domain which is essential for kinase activity and fused this to the 6 × His tag. As shown in Figure 2A, consistent with previous reports [3], CERK1 has very strong *in vitro* kinase activity. In contrast, under the same conditions, LIK1 had a measurable but significantly lower activity. As expected, the LIK1^D798A^ protein did not show any detectable kinase activity, indicating that Asp-798 of LIK1 is critical for its activity. When LIK1 and CERK1 are both present, a strong, labeled His-LIK1 band was detected, suggesting that LIK1 can be phosphorylated by CERK1 *in vitro*. Interestingly, the kinase dead version LIK1D^798A^ was also strongly phosphorylated by CERK1, but the intensity of the band was much less than that seen for wild-type LIK1.

To determine the *in vivo* phosphorylation of LIK1, a phos-tag gel was used to separate phosphorylated LIK1. LIK1 gene fused with 4 × Myc tag was transformed into *Arabidopsis* plants under the control of the *35S* CaMV promoter (Figure S6). As shown in Figure 2B, the levels of phosphorylated LIK1 were much higher in wild-type Col-0 than in the *cerk1* mutant plant, suggesting that phosphorylation of LIK1 is largely dependent on CERK1 protein. However, no difference in the levels of phosphorylated LIK1 was detected before and after chitooctaose treatment, indicating that the phosphorylation of LIK1 is not regulated by chitin treatment.

### *Lik1* mutant plants are more responsive to both chitin and flagellin treatment

**Figure 3:**
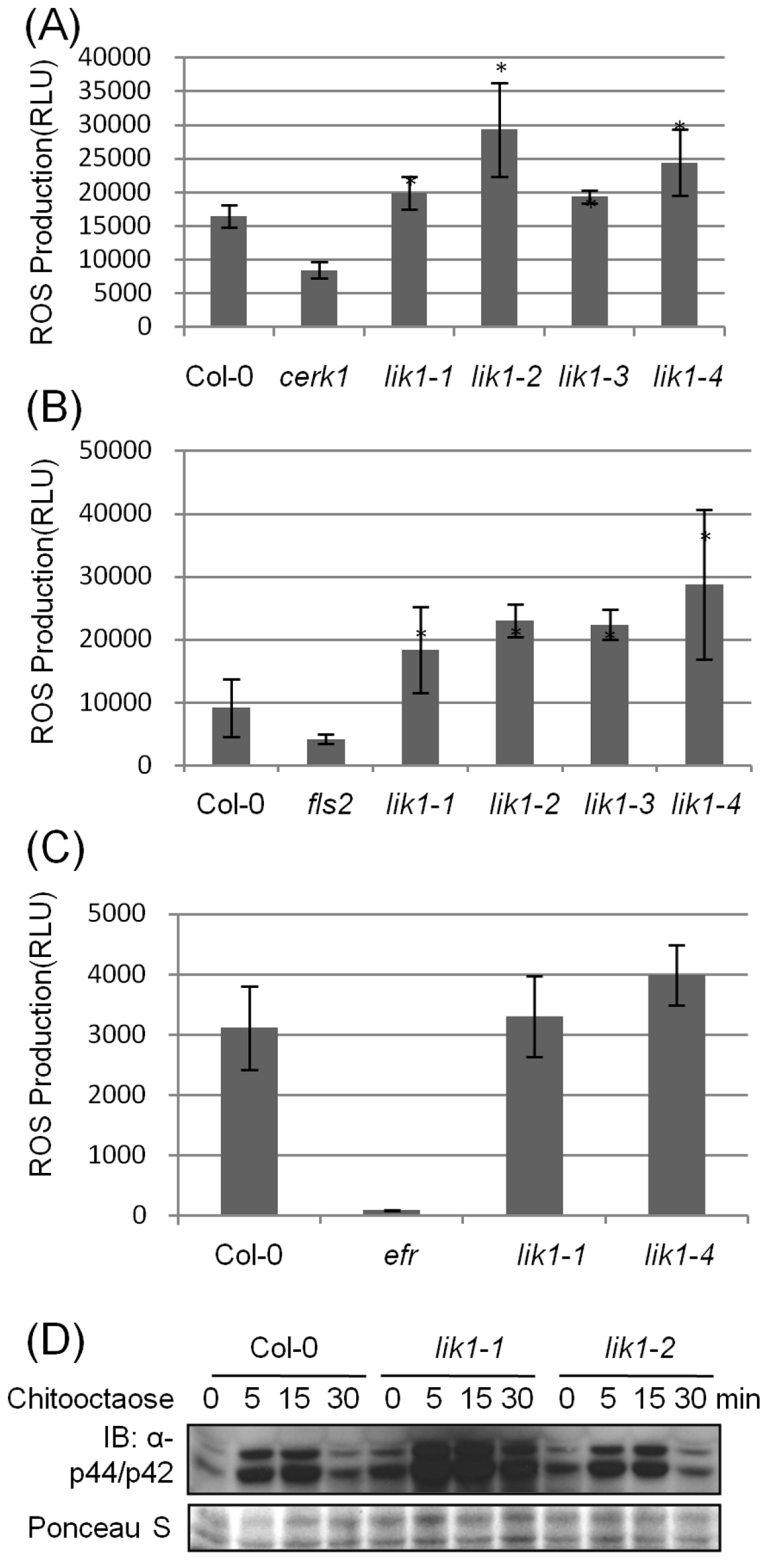
*Lik1* mutants show enhanced responses to chitin elicitor. ROS generation in *lik1* mutant lines treated with chitooctaose (A), flg22 (B) or elf26 (C). ROS was analyzed 20 seconds before and after the maximum signal observed. The data are the average of 12 leaf discs punched from six 4-week-old plants. (Bars represent standard deviations). Student T-test (*)<0.05. The experiment was done in triplicate, each with similar results. D, MPK phosphorylation in mature leaf treated with chitooctaose at the time point indicated in the figure, determined by immunoblotting using anti-MPK3/MPK6 antibody. Lower gel reveals similar loading of total protein using poceau s staining.

*Lik1* T-DNA mutant plants produced significantly more ROS when treated with chitooctaose (Fig 3A and S7A). In addition to chitin treatment, we also tested the response of these mutants to other well-characterized MAMPs, specifically flg22 and elf26. As shown in Figure 3B and S7B, *lik1* mutant plants also showed significantly higher ROS production when treated with flg22. In contrast, the mutants responded similarly to the wild-type when treated with elf26 (Figure 3C). Therefore, LIK1 acts in both the chitin and flagellin signaling pathways as an apparent negative regulator, but not in the response to all MAMPs. To confirm the negative role of LIK1 in chitin-induced immune responses, MPK3/6 phosphorylation was measured after chitin treatment. As shown in Fig 3D, both Col-0 wild-type and *lik1* mutants plants (*lik1-1* and *lik1-2*) showed significantly enhanced phosphorylation of MPK3 and MPK6 after chitin treatment. However, the phosphorylation of MPK3 and MPK6 was significantly increased before chitin treatment in *lik1* mutants, suggesting that LIK1 plays a negative role in mediating chitin response in plants.

### *Lik1* mutant plants are more resistant to the hemibiotrophic pathogen *P. syringae* but more susceptible to the necrotrophic fungal pathogen *S. sclerotiorum*

**Figure 4:**
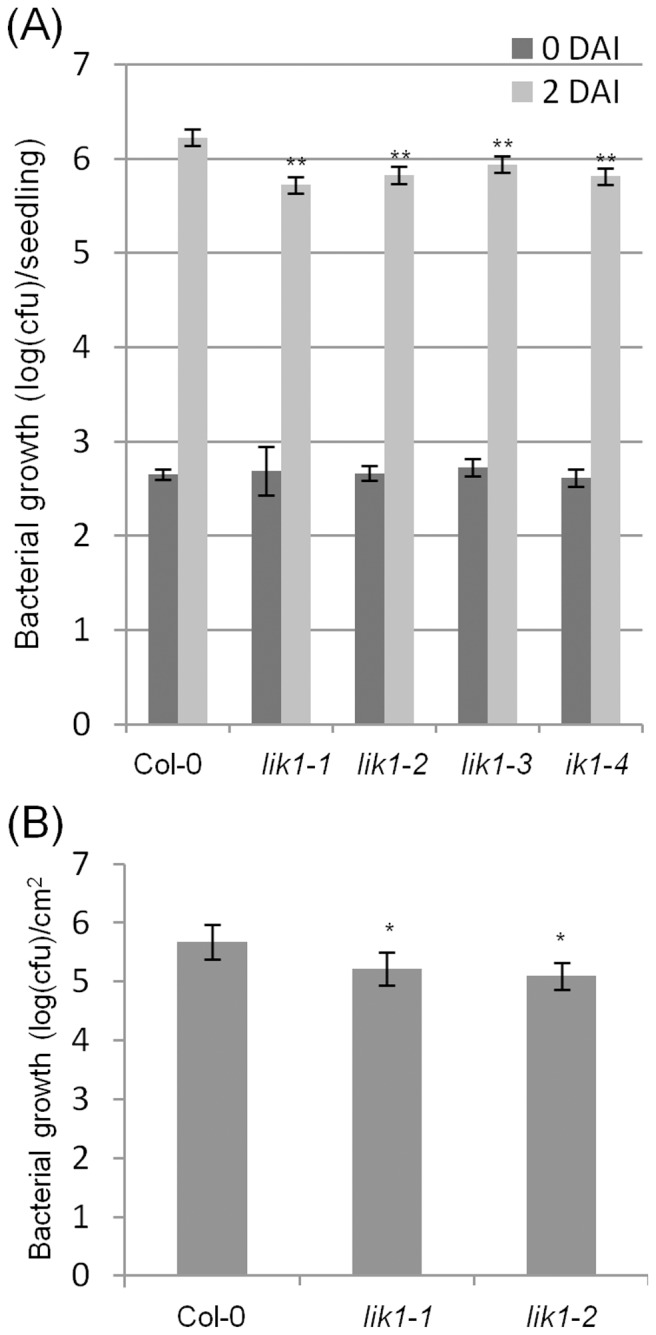
*Lik1* mutant plants are more resistant to *Pseudomonas syringae* pv. *tomato* DC3000. A. Ten-day-old mutant and wild-type seedlings were inoculated with *P. syringae* pv. *Tomato* DC3000 at the concentration of 0.5×10^4^ cfu/ml by soaking in a bacterial suspension for 3 hours. The bacterial solution was then removed and the seedlings were washed three times with H_2_O prior to incubation. Bacterial growth was measured by grinding the seedlings and then plating the resulting extracts on YPD medium with rifampicin and kanamycin as selection. The data are shown as the log10 of colony forming units (3 hours and 48 hours after inoculation) per seedling. The data are the average of 18 seedlings. Bars represent standard deviations. Student T-test (**) P<0.01. The experiment was done in triplicate, each with similar results. B. Leaf populations of *P. syringae* pv. *tomato* strain DC3000 from 4-week-old. Data are mean ±SE for three separate experiments. Student T-test (*) P<0.05.

**Figure 5:**
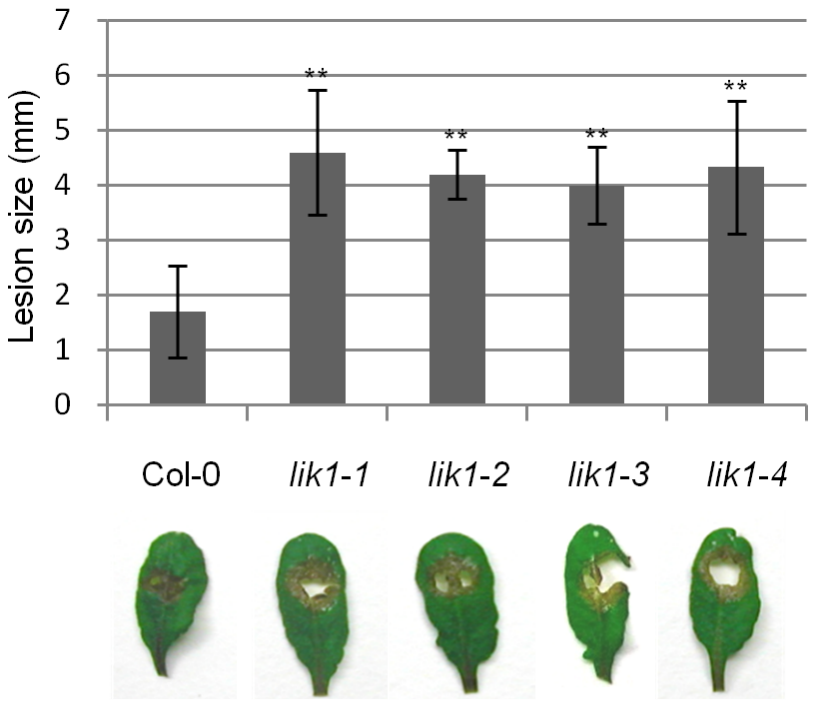
*Lik1* mutant plants are slightly more susceptible to *Sclerotinia sclerotiorum*. Four-week old mature leaves were inoculated with *S. sclerotiorum* by attaching 2 mm diameter agar discs of *S. sclerotiorum* mycelium onto the surface of detached leaves. Lesion size was measured 16 hours after inoculation. The data represent the average lesion size of 18 leaves from 6 plants. The experiments were performed in triplicate, each with similar results. Bars represent standard deviations. Student T-test (**) P<0.01. The pictures were taken 24 hours after inoculation. The experiment was done in triplicate, each with similar results.

The data above clearly indicate that LIK1 is involved in both fungal chitin and bacterial flagellin responses. Therefore, wild-type and mutant plants were challenged with the hemibiotrophic, bacterial pathogen *P.syringae* pv. *tomato* DC3000 and the necrotrophic, fungal pathogen *S. sclerotiorum.* Figure 4 shows that bacterial growth was reduced in *lik1* mutant plants in both young seedlings (Figure 4A) and mature leaves (Fig 4B), indicating that LIK1 functions as a negative regulator of the defense pathways responding to hemibiotrophic *P. syringae* pathogens. In contrast, *lik1* mutant plants displayed enhanced disease susceptibility to *S. sclerotiorum*, as indicated by the larger lesion sizes (Figure 5), suggesting that LIK1 acts as a positive regulator of defense pathways against necrotrophic pathogens.

### *Lik1* mutant plants are defective in the expression of genes involved in the JA/ET signaling pathways

**Figure 6:**
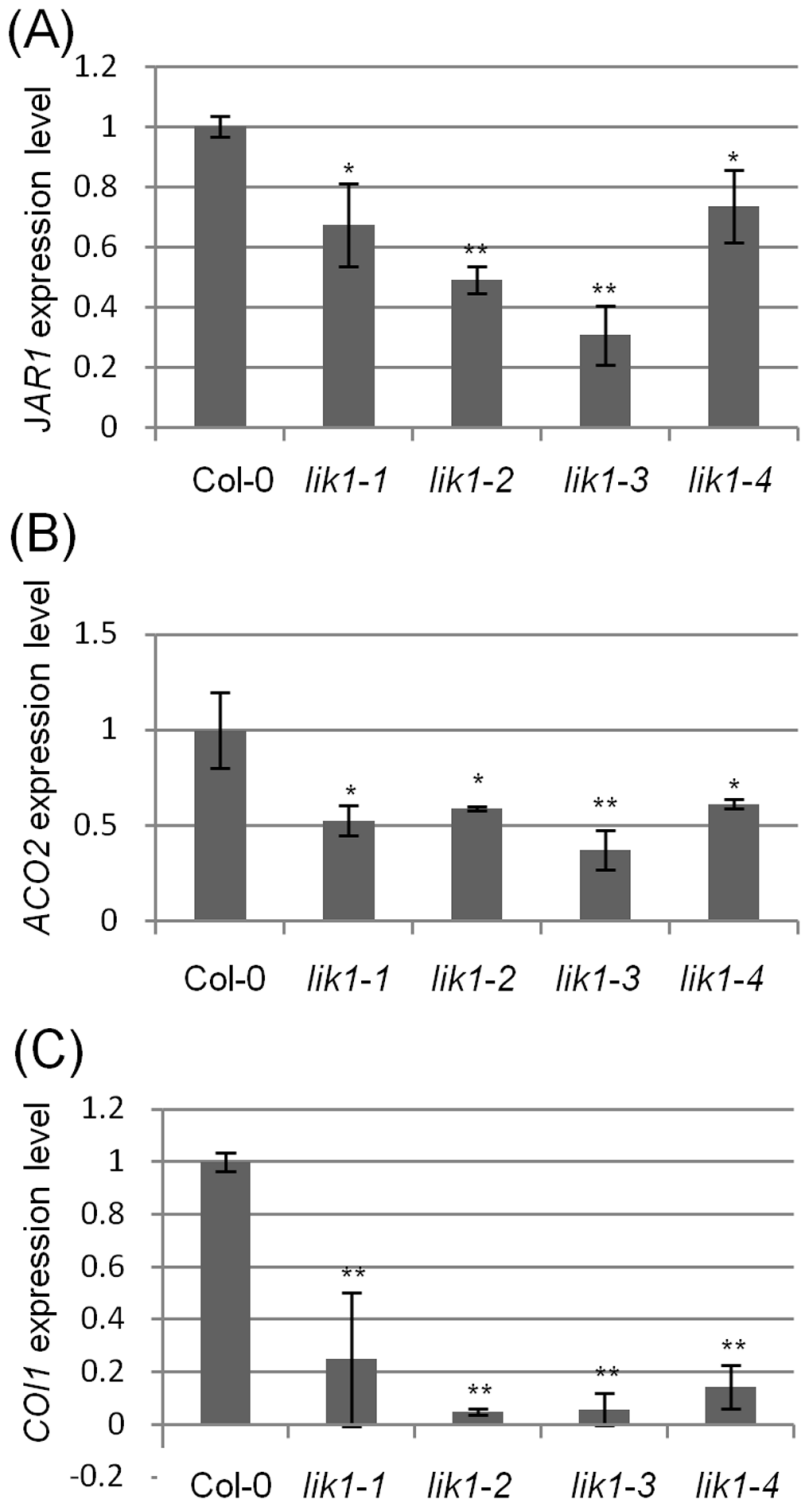
*Lik1* mutant plants show reduced expression of key genes involved in the JA/ET pathways. RNA isolated from 10-day-old seedlings were used to quantify gene expression by qRT-PCR. The genes chosen represent key genes (as shown in Figure) in the JA (A and C) and ET (B) signaling pathways (Table S2). The data represent the average of the ratio of gene expression in the mutants compared to the wild-type for three biological replicates. Bars represent standard errors. Student T-test (*) P<0.05, (**) P<0.01.

Two parallel hormone pathways are known to mediate distinct mechanisms of defense against either hemibiotrophic or necrotrophic pathogens. The salicylic acid (SA)-dependent signaling pathway primarily targets biotrophs and hemibiotrophs; whereas the jasmonic acid (JA) and ethylene (ET)-dependent signaling pathways primarily mediate resistance to necrotrophic pathogens [21]. As mentioned above, analysis of the *lik1*mutant lines suggests that LIK1 normally suppresses the SA pathway (consistent with the greater resistance of the mutants to *P. syringae*) and activates JA and/or ET pathways (consistent with the greater susceptibility of the mutants to *S. sclerotiorum*). In order to test this hypothesis, the expression of key genes in the SA and JA/ET pathways was examined in wild-type and *lik1* mutant plants either mock-treated or treated with chitooctaose. Figure 6 shows that the expression of key JA/ET signaling pathway genes, *JAR1*, *ACO2* and *COI1,* as measured by quantitative real-time-PCR (qRT-PCR). Expression of *JAR1*, a gene involved in formation of JA-amino acid conjugates [22], is low in *lik1* mutants (Fig 6A), indicating a suppression of the JA signaling pathway in the mutant. *ACO2* encodes ACC oxidase 2, a vital enzyme involved in ET synthesis. In *lik1* mutant plants, this gene is expressed at a consistently lower level relative to wild-type plants (Fig 6B). COI1 is a co-receptor for JA [23]. In *lik1* mutant plants, *COI1* expression was consistently lower than in the wild-type plants (Fig 6C). In contrast to these results, measurements of gene expression of key SA pathway genes, such as *PR1*, *PR2* and *SID2*, showed no effect of the *lik1* gene mutations (data not shown). Therefore, the data support the postulated role of LIK1 as a positive regulator of the JA and ET pathways.

## Discussion

### LIK1 negatively regulates MAMP-triggered innate immunity

The data presented support the idea that LIK1 is an important component of AtCERK1-mediated chitin perception and negatively regulates, chitin-triggered innate immunity. LIK1 directly interacts with AtCERK1 as demonstrated by Yest two hybrid and co-immunopreciptation. *In vitro* and *in vivo* experiments indicate that phosphorylation of LIK1 is dependent on CERK1. Interestingly, the interaction between LIK1 and CERK1 is reduced 30 min after chitin perception by CERK1. The absence of LIK1 in *lik1* mutant plants also leads to increased ROS production and an enhanced response to chitin elicitation. These data suggest that LIK1 and CERK1 may transiently interact, as part of the early events in chitin recognition. Subsequently, LIK1 may disassociates from CERK1, perhaps as a necessary step for further chitin signaling and triggering defense responses. Consistent with the negative role in chitin-triggered immunity, mutations in *LIK1* strongly affect plant resistance to the necrotrophic pathogen *S. sclerotiorum*, as well as the hemibiotrophic pathogen *P. syringae* pv. *tomato* DC3000. Given the lack of chitin in bacteria, the latter result may be viewed as surprising. However, Willmann et al [12] demonstrated that AtCERK1 is also a critical component, in conjunction with the LysM proteins, LYM1 and LYM3, of a peptidoglycan receptor complex that acts to trigger plant defense to bacterial pathogens.

However, we were surprised to find that mutations in *LIK1* affected not only chitin signaling but also the response to flg22, derived from the bacterial flagellin elicitor. Although the mechanism of this effect is still unclear, it may be mediated through protein components that are common to both the AtFLS2 and AtCERK1 receptor complex (e.g., BIK1, see below) or downstream signaling pathways. For example, there is compelling evidence that the MAMP signaling pathways share many downstream steps [2], [24], [25], including the MAPK cascade [26] and WRKY transcription factors [27], converging on a largely common set of MAMP induced genes (e.g., 441 genes commonly up-regulated by chitin, flg22 and elf18) [2].

Several proteins have now been identified as co-receptors or components of MAMP signaling receptor complexes. For example, the brassinosteroid-associated kinase 1 (BAK1), initially identified as a protein interacting with BRI1, the receptor of the phytohormone brassinosteroid [28]–[30]), interacts with the flagellin receptor, FLS2, and is required for flagellin-induced immunity [31]–[33]. Although both BAK1 and LIK1 are membrane associated LRR-RLKs with association with MAMP receptors, the function of these two proteins is not the same. The association between BAK1 and FLS2 requires flagellin elicitation[31], [32], However, unlike LIK1, BAK1 plays a positive role in MAMP-triggered immune responses since mutant plants defective in BAK1 and BAK1-like1 lose the response to both flagellin and EF-Tu, including decreased ROS and ethylene production, as well as a reduction in defense gene expression [34]. In contrast, LIK1 associates with CERK1 before and after chitin treatment, albeit this is transient showing a marked reduction 30 min after treatment. Also, in contrast to BAK1, LIK1 is a negative regulatory of chitin signaling. Therefore, we propose that LIK1 is a novel component involved in both chitin and flagellin-triggered immunity in plants. The exact mechanism of LIK1 action remains to be determined but the current data does suggest a possible model. In this model, LIK1 negatively impacts chitin perception by CERK1 either as a direct inhibitor, a decoy that, for example, misdirects phosphorylation to itself rather than downstream components, or through modifying the interaction with other co-receptors. Regardless of the mechanism of inhibition, this inhibition would likely be relieved by dissociation of LIK1 from CERK1 shortly after chitin binding. Indeed, LIK1 interaction with CERK1, prior to chitin recognition, may act to maintain the CERK1 receptor in an inactive state to prevent precocious induction of innate immunity pathways, which is known to be detrimental to plant growth [35].

### LIK1 is a positive regulator of the JA/ET hormone signaling pathway

Mutations in *LIK1* differentially affect plant susceptibility to hemibiotrophic and necrotrophic pathogens. The plants are more resistant to the former and more susceptible to the latter. Consistent with this finding, *lik1* mutant plants showed a significantly lower expression of key genes involved in the JA/ET signaling pathways. Therefore, we hypothesize that LIK1 is a positive regulator of these pathways. Given that the JA/ET and SA pathways often act antagonistically [36], the lower expression of the JA/ET pathway may explain the greater resistance to *P. syringae*. However, we detected no change in the expression levels of key SA pathway genes. Consistent with this, it should be noted that the differences between the wild-type and *lik1* mutants challenged with *P. syringae* were not as dramatic as the response to the necrotrophic fungus *S. sclerotiorum*.

### The increase in ROS production in *lik1* mutants may promote infection by necrotrophic fungi

Although ROS production has antimicrobial activity [37], [38], excessive ROS production can also facilitate necrotrophic infection since it promotes programmed cell death [39]. This hypothesis would be consistent with the finding that *lik1* mutants generated significantly more ROS upon MAMP treatment and showed an increased susceptibility to the necrotrophic fungal pathogen *S. sclerotiorum*.

## Materials and Methods

### Yeast Two Hybrid Screening

The region containing the intracellular low complexity and kinase domain of AtCERK1 was amplified from cDNA derived from *Arabidopsis* Col-0 plants and cloned into pGEM-T easy using T-A cloning according to the manufacturer's protocol (Promega, Madison, WI). This DNA fragment was then digested with *Bam*H I and *Eco*R I, and the resulting fragment was cloned into the pEG202 bait vector (OriGene, Rockville, MD). The primers used for verification of the cloning are indicated in Table S1.

The bait vector was transformed into yeast EGY194 strains harboring each of four different reporter plasmids to test for bait self-activation and nuclear localization following the DupLEX-A yeast transformation protocol (OriGene, Rockville, MD). The prey library was created from 10-day-old seedlings pretreated with 100 ng/ml chitin for 30 minutes under the construction of the DupLEX-A yeast-two hybrid system pJG4-5 vector (OriGene, Rockville, MD). A total of 100 µg of the prey library cDNA was transformed into the yeast strain EGY194 harboring the bait vector and the reporter plasmid pJK103 following the DupLEX-A yeast two-hybrid system protocol (OriGene, Rockville, MD). The transformation mixture was plated on YNB medium (OriGene, Rockville, MD) with galactose, but lacking uracil (the reporter gene plasmid marker), histidine (the bait plasmid marker) and tryptophan (the library plasmid marker). The plates were incubated at 30°C and examined after 4 to 5 days. Colonies growing on the medium were purified by streaking onto YNB medium lacking selection nutrients (OriGene, Rockville, MD).

Plasmids from positive colonies were isolated from yeast using a Zymoprep II-yeast plasmid miniprep kit (Zymo, Irvine, CA). The purified plasmids were subsequently heat-shock transformed into *E. coli* strain KC8, which was grown on M9 glucose medium (OriGene, Rockville, MD) lacking tryptophan to eliminate the bait vector and to selectively recover each cDNA-containing plasmid. Each positive clone was then re-transformed into a yeast strain EGY194 harboring the bait and reporter gene plasmid (pJK103) and again screened on selective medium to confirm interaction. Confirmed prey plasmids were then sequenced to characterize the positive clones.

### Plant germination and growth conditions

*Arabidopsis thaliana* seeds were surface-sterilized with 70% ethanol, incubated in a solution containing 3% sodium hypochlorite and 0.2% Tween-20 for 5 minutes, washed five-times with sterile H_2_O and finally re-suspended in 300 µl sterile water. Seeds were then vernalized at 4 °C for 3 days in the dark and germinated on solid 1% agar of 0.5×MS medium containing Murashige and Skoog mineral salts [40] (Sigma, St. Louis, MO), 0.5% sucrose (w/v), 0.05% MES (pH 5.7) and 0.7% agar (Fisher Scientific). For seedling experiments, plants were grown on 0.5×MS agar medium for 3 days and transferred into a liquid 0.5×MS medium. Otherwise, seedlings were grown for up to 2 weeks prior to transfer to soil. Plants were grown in a plant growth chamber (model CU-32L, Percival Scientific Inc., Boone, IA) under 8 hour day/16 hour night cycle and 60% humidity. For seed amplification and analysis of mature plants, 14-day-old seedlings were transferred to Pro-mix soil (Premier Horticulture, Red Hill, PA) and grown at 22 °C under continuous fluorescent white light in either a plant growth chamber with 60% humidity or in a greenhouse.

### MAMP treatments

*Arabidopsis* 14-day old seedlings or 5-mm diameter 4-week old leaf discs were soaked into the different MAMP solutions with the following concentrations used for each MAMP: 100 µg/ml chitin mixture (chitin from crab shells, Sigma, St Louis, MO), 1 µM chitooctaose (Sigma, St Louis, MO), 1 µM elf26, 1 µM flg22 (GenScript, Piscataway, NJ).

### Reactive oxygen species assay

*Arabidopsis* 14-day-old seedlings or 5-mm diameter 4-week-old leaf discs were incubated in H_2_O in a growth chamber overnight to recover from the wound response. One hour prior to each experiment, plant tissues were transferred to the dark (to suppress the light background arising from photon emission from the plastic plates that could be detected by the camera) and treated with a fresh solution containing the designated MAMP, 35 µg/ml luminol solution and 20 µg/ml horseradish peroxidase (Sigma, St. Louis, MO). Immediately after treatment, the ROS signal was recorded for 10 minutes; an interval 20 seconds before and 20 seconds after the maximum signal observed was analyzed using a Photek camera PSU1 (Photek ltd., San Francisco, CA).

### Pathogen assays

*P. syringae* pv. *tomato* DC3000 *lux DCABE* [41] bacterial cultures were grown on NYG medium (Fisher Scientific) plates for 24 hours, at 30°C prior to inoculation onto plants. Antibiotics were used for plate selection at the following concentrations: 25 µg/ml kanamycin and 100 µg/ml rifampicin (Fisher Scientific). For MAMP treatment, 10-day old seedlings were treated with MAMPs by soaking seedlings in the MAMP solution for 24 hours. Similarly, seedlings were soaked in the bacterial solution at a concentration of 0.5×10^4^ cfu/ml for two hours. The bacterial solution was then removed and seedlings were washed with H_2_O three times. After 72 hours of inoculation, plants were surface-sterilized for 30 seconds with 70% ethanol. The bacterial extract was plated onto NYG plates with selection antibiotics and grown at 30°C for counting after 48 hours. In another procedure, bacterial growth was quantified using a Photek PSU1 camera to detect luminescence 24 hours after inoculation.

Four-week-old *Arabidopsis* seedlings were inoculated with *P. syringae pv. tomato* strain DC3000 at OD_600_ = 0.0001 in 10 mM MgCl_2_ solution. Leaf discs were taken from four inoculated rosette leaves and ground in 10 mM MgCl_2_ 3 days after inoculation. Samples were ground and plated on NYGA plates with 25 mM rifampicin, and colony counts were recorded two days after incubation at 28 °C.

*S. sclerotiorum* cultures were grown on potato dextrose agar medium as described in the method of Dickman and Mitra [42]. Two leaves were detached from 4-week-old *Arabidopsis* plants (with six biological replicates for each genotype) and transferred onto damp Whatman paper in a petri dish. A 2 mm-diameter agar disc of *S. sclerotiorum* mycelium was then placed upside down onto each leaf. Eighteen hours after inoculation, the fungal disc diameters were measured at 4 different angles for each disc and the average size was recorded.

### Total RNA isolation and quantitative reverse transcription polymerase chain reaction (qRT-PCR)

Total RNA was isolated from 10-day-old seedlings of *Arabidopsis* using the Trizol reagent (Invitrogen, Carlsbad, CA) following the manufacture's protocol. RNA concentrations were measured using a Nanodrop-1000 spectrophotometer. RNA quality was assessed by agarose gel electrophoresis. RNA samples were purified using the Qiagen RNeasy Mini Kit (Qiagen, Valencia, CA) according to the manufacturer's protocol. To remove genomic DNA contamination, total RNA was treated with TURBO DNase (Ambion Inc., Houston, TX) according to the manufacturer's protocol. Two micrograms of RNA were reverse-transcribed to synthesize single-stranded cDNA using Superscript III reverse transcriptase (Invitrogen, Carlsbad CA), oligo (dT), 10 mM dNTP and RNase Out at 37 °C for 1 hour in a 25 µl reaction. The reaction was inactivated by heating at 70 °C for 15 minutes.

In order to measure *LIK1* gene expression in the T-DNA insertion mutant lines, 1 µl of RT-PCR product was used for PCR with Ex-Taq DNA Polymerase (Takara, Otsu, Shiga, Japan) and gene-specific primers as listed in Table S1. As an internal control, gene-specific primers of *ubiquitin 5* (UBQ5) were used together with specific primers in each PCR reaction. To analyze expression of specific genes, 6 µl of the PCR reaction was used for agarose gel electrophoresis.

Quantitative RT-PCR was performed with an ABI 7500 Real Time PCR System with Sequence Detection System (SDS) software version 1.3 (Applied Biosystems, Foster City, CA) utilizing SYBR Green PCR Master Mix (Applied Biosystems, Foster City, CA). The gene-specific primers utilized are listed in Table S2. PCR conditions were as follows: stage 1, 50°C for 2 min; stage 2, 95°C for 10 min, stage 3, 45 cycles of 95°C for 15 sec and 60°C for 1 min; stage 4 (for dissociation), 95°C for 15 sec, 60°C for 1 min, and 95°C for 15 sec. Data obtained for three technical replicates of each biological replicate were normalized to AT2g28390 (SAND), a gene that is constitutively expressed under the culture conditions used [43]. Gene expression levels were calculated by efficiency^(-ΔCT)^ where ΔCT is calculated by subtracting the cycle threshold (CT) of the reference gene from CT of the each gene of interest, and then converted as relative values to the gene expression level at the starting time of the treatment (initial (0 min) = 1.0). Primers used were listed in Table S2.

### Recombinant protein purification and kinase assay

DNA fragments encoding the AtCERK1 and AtLIK1 kinase domains were amplified by PCR and inserted into pGEX5X-1 and pET-22b at the *Bam*H I and *Xho* I sites, respectively. The point mutation D798A of AtLIK1 was made using plasmid pET-*LIK1*and a site-directed mutagenesis kit (Stratagene, La Jalla, CA). The plasmid pGEX-*CERK1* was transformed into BL21 (AI), while the plasmid pET-LIK1 was transformed into BL21 (DE3) expressing the YopH tyrosine phosphatase [44]. Protein production was induced by adding 0.1 mM isopropyl β-D-thiogalactopyranoside (IPTG) when the culture reached an OD600 of 0.6. The protein was extracted after inducing the cultures for 24 hours at 16 °C. Recombinant protein purification was done according to the following procedure. Bacterial cells from ∼200 ml LB medium were pelleted by centrifugation at 4 °C at 8,000 rpm for 10 min and resuspended in 10 ml 1×PBS solution supplemented with 1×EDTA-free protease inhibitor (Roche, Indianaplois, IN), 0.5% Triton X-100, 1 mg/ml lysozyme and put on ice for 30 min. The cells were lysed by sonication before centrifuging at 13,000 rpm for 10 minutes at 4 °C. Supernatants were used for protein affinity purification using Glutathione Sepharose 4B (GE Healthcare, Milwaukee, WI) or TALON Cobalt Resin (ClonTech, Mountain View, CA). The column was washed with at least 20 bed volumes of 1× PBS solution. The eluted proteins, including GST, GST-CERK1, His-LIK1, and His-LIK1^D798A^, were dialyzed with buffer (50 mM Tris (PH 7.5), 50 mM KCl, 2 mM DTT, and 10% glycerol).

The *in vitro* kinase assays used 1 µg purified protein in a buffer containing 50 mM Tris (PH 7.5), 50 mM KCl, 2 mM DTT, 5 mM MnCl_2_, 5 mM MgCl_2_, 10% glycerol, 10 µM ATP and 5 µM Ci [γ-^32^P]-ATP. The assay mix was incubated at 28 °C for 30 minutes and the reaction was stopped by adding 1×SDS loading buffer. The samples were separated on 12% SDS-PAGE gel and the gel imaged by autoradiography using phosphor screens and a phosphorimager.

For *in vivo* phosphorylation assay, 40 µM Phos-tag reagent (AAL-107, FMS Laboratory) was used to separate phosphorylated LIK1 according to the manufacture's manual. 10-day-old transgenic seedlings expressing *LIK1-Myc* in both Col-0 and *cerk1*mutant backgrounds were treated with 1 uM chitooctaose for the times shown in Figure 3B. For the phosphatase treatment, the antarctic phosphatase (New England Biolabs, Ipswich, MA) was used according to the protocol at 37°C for 15 min.

### Protein Co-immunoprecipitation

Full-length *AtCERK1* and *AtLIK1*genes were amplified by PCR from *Arabidopsis* genomic DNA and cloned into pDONR-Zoe vector using the BP reaction (Invitrogen). The resultant plasmids were then recombined using the LR reaction into pUC-GW14 and pUC-GW17 [45], respectively. *Arabidopsis* protoplasts were isolated from about 8-week old Col-0 wild-type plants grown under short day conditions (8-hour light/16-hour dark) according to the protocol described by Yoo, et al [46]. For co-immunoprecipitation, ∼1 ml of protoplasts (∼10^6^ cells) were transfected with 100 µg plasmids, and the transfected protoplasts were incubated at room temperature for 14 hours. Chitin mixture (1 ul of a 100 µg/ml chitin mixture solution) was added to 1 ml of the protoplast solution before harvest for lysis. Transfected protoplasts were lysed with 0.5 ml protein extraction buffer (50 mM Tris (PH 7.5), 150 mM NaCl, 0.5% Triton X-100, and 1×Cocktail protease inhibitor (Sigma, St. Louis, MO) for 30 minutes on ice. The resulting extract was centrifuged at 14,000 rpm for 15 minutes at 4 °C. The clear supernatant was then added with 2 µl anti-Myc (Covance, Princeton, NJ) and incubated at 4 °C for 3 hours, 20 µl pre-equilibrated protein A agarose was added for 1 hour and shaking gently at 4 °C using a reciprocal shaker. The agarose beads were washed with 1 ml of buffer (50 mM Tris (pH 7.5), 150 mM NaCl) at least 4 times. Protein was eluted with 50 µl 1×SDS loading buffer by heating at 80 °C for 10 min. The eluted protein and input protein were separated on 10% SDS-PAGE gel and transferred to PVDF membrane for Western blot analysis. Antibodies HA-peroxidase (Roche, Indianapolis, IN) and anti-Myc (ab9106, Abcam, Cambridge, MA) were diluted at 1∶1000 and 1∶3000, respectively, for western blots. Both signals were detected using SuperSignal West Pico and Femto chemiluminescent substrate (Thermo Scientific, Rockford, IL) and captured with a FujiFilm LAS-3000 (FujiFilm, Minato-ku, Tokyo, Japan).

### MPK phosphosrylation assay

1 uM Chitiooctaose was hand-infiltrated into leaf from Col-0 wild-type and *lik1* mutant plants for the times indicated in the Figure. Crude protein was extracted in a buffer containing (50 mM Tris (PH 7.5), 150 mM NaCl, 0.5% Triton X-100, and 1×Cocktail protease inhibitor (Sigma, St. Louis, MO) and separated on SDS-PAGE gel. After transferred on PVDF membrane, phosphorylated MPK3 and MPK6 were detected using anti-P44/P42 antibody (Cell Signaling Technology, Beverly, MA).

### *Arabidopsis* transformation

Full-length genomic DNA of *AtLIK1* was recombined into pGWB17 binary vector [47]. The resultant plasmid was electroporated into *Agrobacteium tumefaciens* GV3101 (pMP90) and transformed into Col-0 wild type and *cerk1* mutant plants by floral dipping method [48].

## Supporting Information

Figure S1 **Procedure of Y2H screening for proteins interacting with the AtCERK1 kinase domain.**
(PDF)

Figure S2 **T-DNA mutants with altered chitin-induced ROS production.**
(PDF)

Figure S3 ***P. syringae*** **pv.** ***Tomato*** **DC3000 growth on 10-day-old seedling.**
(PDF)

Figure S4 **Analysis of** ***lik1*** **insertion mutants.**
(PDF)

Figure S5 **Second example (see also** **Figure 1A** **) of association between CERK1 and LIK1 in protoplasts.**
(PDF)

Figure S6 **Expression of** ***LIK1*** **in transgenic** ***Arabidopsis*** **Col-0 and** ***cerk1*** **mutant plants determined by western blotting.**
(PDF)

Figure S7 ***Lik1*** **mutants with enhanced ROS production in responses to chitin and flg22 elicitors.**
(PDF)

Table S1 List of 54 proteins interacting with the AtCERK1 kinase domain as identified by LexA-Y2H screening.
(XLSX)

Table S2 Primer sequences.
(XLSX)
