# Supplementary material for: LIK1, A CERK1-Interacting Kinase, Regulates Plant Immune Responses in Arabidopsis
Source: PLoS One. 2014 Jul 18;9(7):e102245. doi: 10.1371/journal.pone.0102245 (PMC4103824; doi:10.1371/journal.pone.0102245)
Supplement: Figure S1 — Procedure of Y2H screening for proteins interacting with the AtCERK1 kinase domain. (PDF) [file pone.0102245.s001.pdf]

(A)

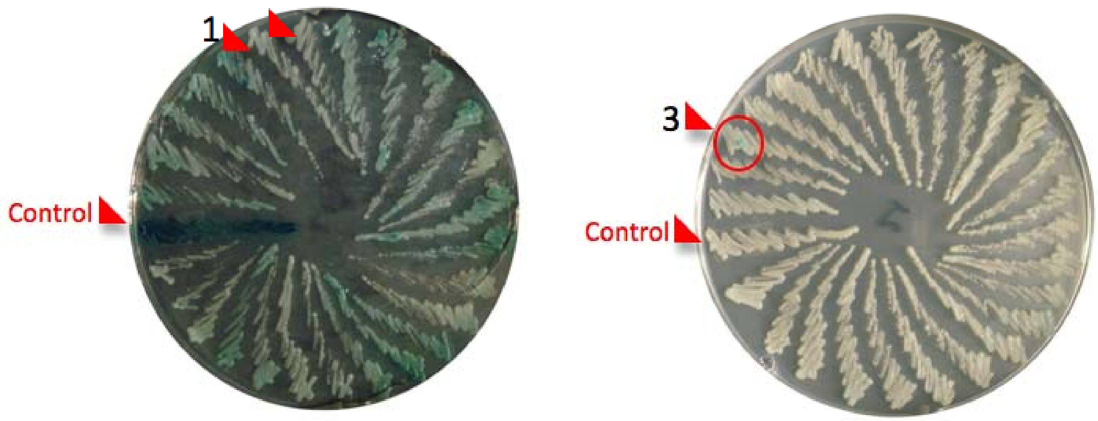

(B)

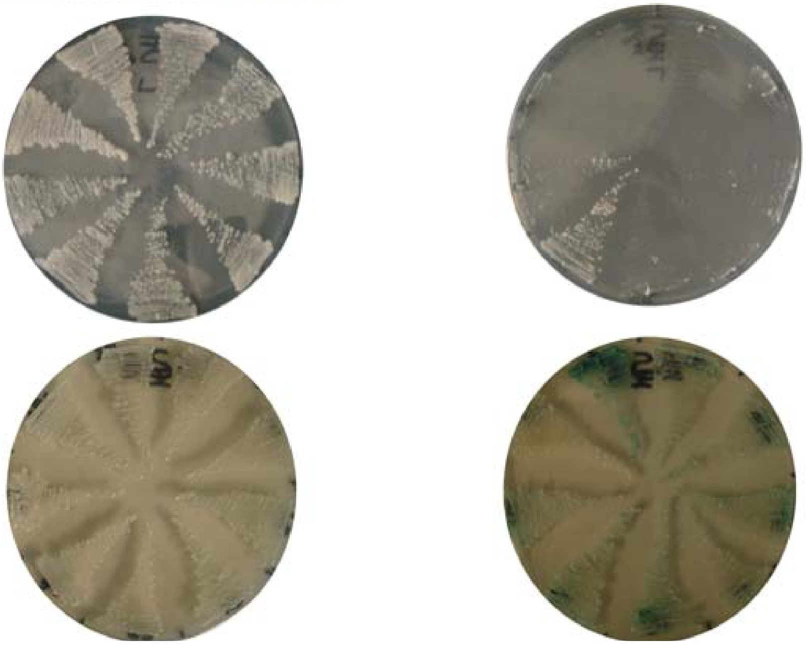

(C)

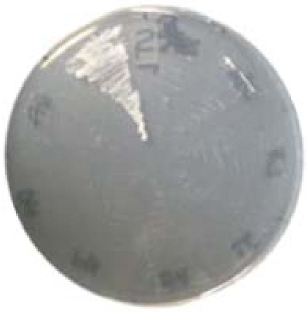

Figure S1. Procedure of Y2H screening for proteins interacting with the AtCERK1

(A) Testing for interaction with the galactose-dependent *lacZ* reporter gene

Colonies growing on selection medium Gal(UTHL)$^-$ were streaked onto selection medium Gal-X gal-(UTH)$^-$ (left panel) and Glc-X gal (UTH)$^-$ (right panel) to test for the ability to activate galactose-dependent LacZ expression. Images were acquired one week after colony streaking. Eg. (1) and (2) did not turn blue on the Gal-X gal-(UTH)$^-$ medium, indicating that there is no protein interacting with the kinase domain of CERK1 (K1) to activate LacZ expression. In addition, (3) is a false positive clone because the colony turned blue on X gal medium regardless of carbon source (the *lacZ* gene is under a Gal promoter).

(B) Testing of individual interactors with CERK1

Individual plasmids were re-transformed into the EGY48 strain containing the bait and reporter genes. Colonies growing on Gal(UTHL)$^-$ were streaked onto selection medium (From left to right, top to bottom Gal(UTHL)$^-$, Glc(UTHL)$^-$, Gal-Xgal-(UTH)$^-$ and Glc-Xgal-(UTH)$^-$).

(C) Testing for self-activation of putative interactors

Confirmed putative interactor plasmids were transformed into EGY48 containing reporter genes. Colonies growing on (UT)$^-$ medium were streaked onto Gal (UTL)$^-$ medium. The images were acquired one week after plating.
