# Supplementary material for: LIK1, A CERK1-Interacting Kinase, Regulates Plant Immune Responses in Arabidopsis
Source: PLoS One. 2014 Jul 18;9(7):e102245. doi: 10.1371/journal.pone.0102245 (PMC4103824; doi:10.1371/journal.pone.0102245)
Supplement: Figure S2 — T-DNA mutants with altered chitin-induced ROS production. (PDF) [file pone.0102245.s002.pdf]

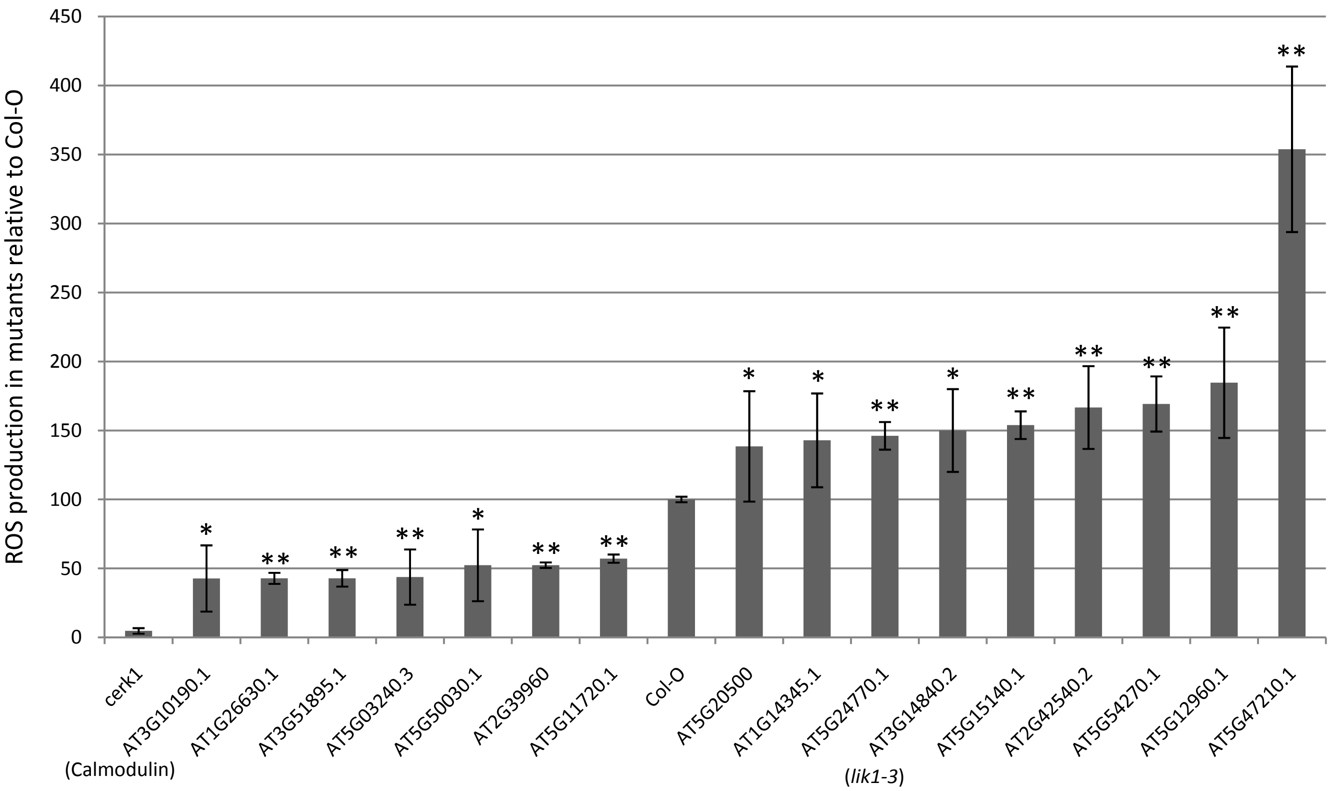

Figure S2. T-DNA mutants with altered chitin-induced ROS production.

ROS production by selected T-DNA insertion mutants after treatment with chitin. ROS production was measured 20 seconds before and after the maximum signal recorded. The data are the average ratio (%) of the ROS signal from 16 seedlings of each mutant compared with the Col-O wild type (100%). These experiments were performed three times, and each replicate gave similar results. Bars represent standard deviations. Student T-test (*) P<0.05, (**) P<0.01.
