# Supplementary material for: LIK1, A CERK1-Interacting Kinase, Regulates Plant Immune Responses in Arabidopsis
Source: PLoS One. 2014 Jul 18;9(7):e102245. doi: 10.1371/journal.pone.0102245 (PMC4103824; doi:10.1371/journal.pone.0102245)
Supplement: Figure S3 — P. syringae pv. Tomato DC3000 growth on 10-day-old seedling. (PDF) [file pone.0102245.s003.pdf]

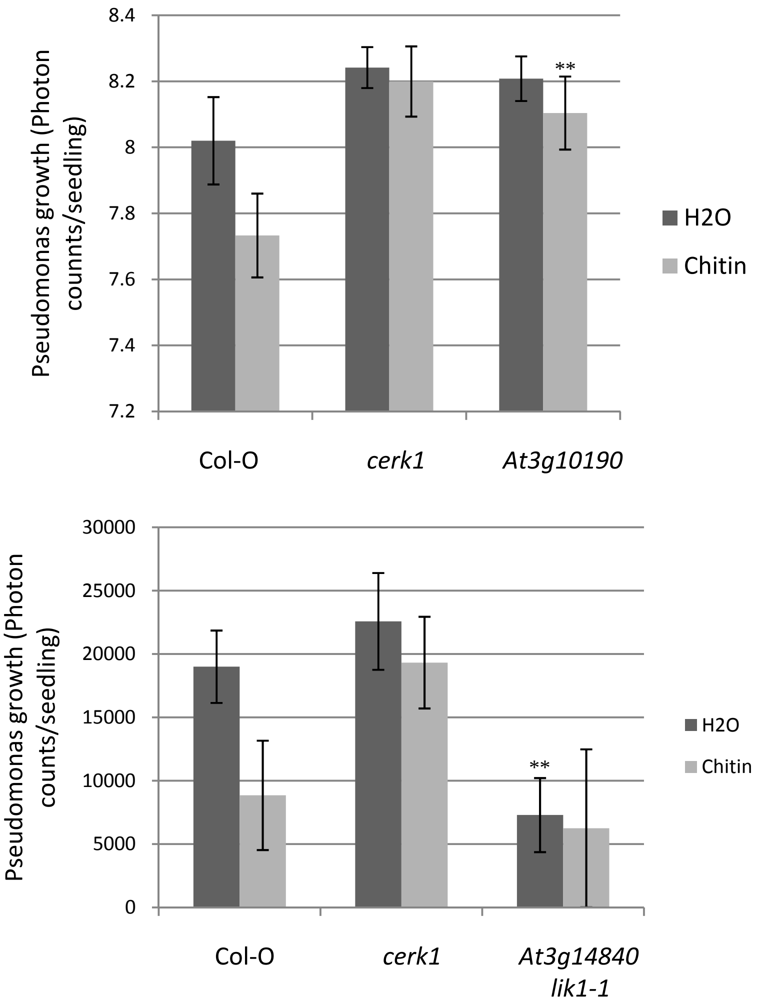

Figure S3. ***P. syringae*** **pv.** ***tomato*** **DC3000 growth on 10-day old seedling.**

10-day old seedlings were pre-treated with chitin for 24 hours, or water as control. Seedlings were inoculated with DC3000 lux DCABE. The luminescent signal was measured 24 hrs after inoculation at intervals of 40 seconds. The data represent the average Lux signal recorded from 16 seedlings. These experiments were performed three times, and each replicate gave similar results. Bars represent standard deviations. Student T-test compared between mutants and Col-O wild type in the same treatment (**) P<0.01.
