# Supplementary material for: LIK1, A CERK1-Interacting Kinase, Regulates Plant Immune Responses in Arabidopsis
Source: PLoS One. 2014 Jul 18;9(7):e102245. doi: 10.1371/journal.pone.0102245 (PMC4103824; doi:10.1371/journal.pone.0102245)
Supplement: Figure S4 — Analysis of lik1 insertion mutants. (PDF) [file pone.0102245.s004.pdf]

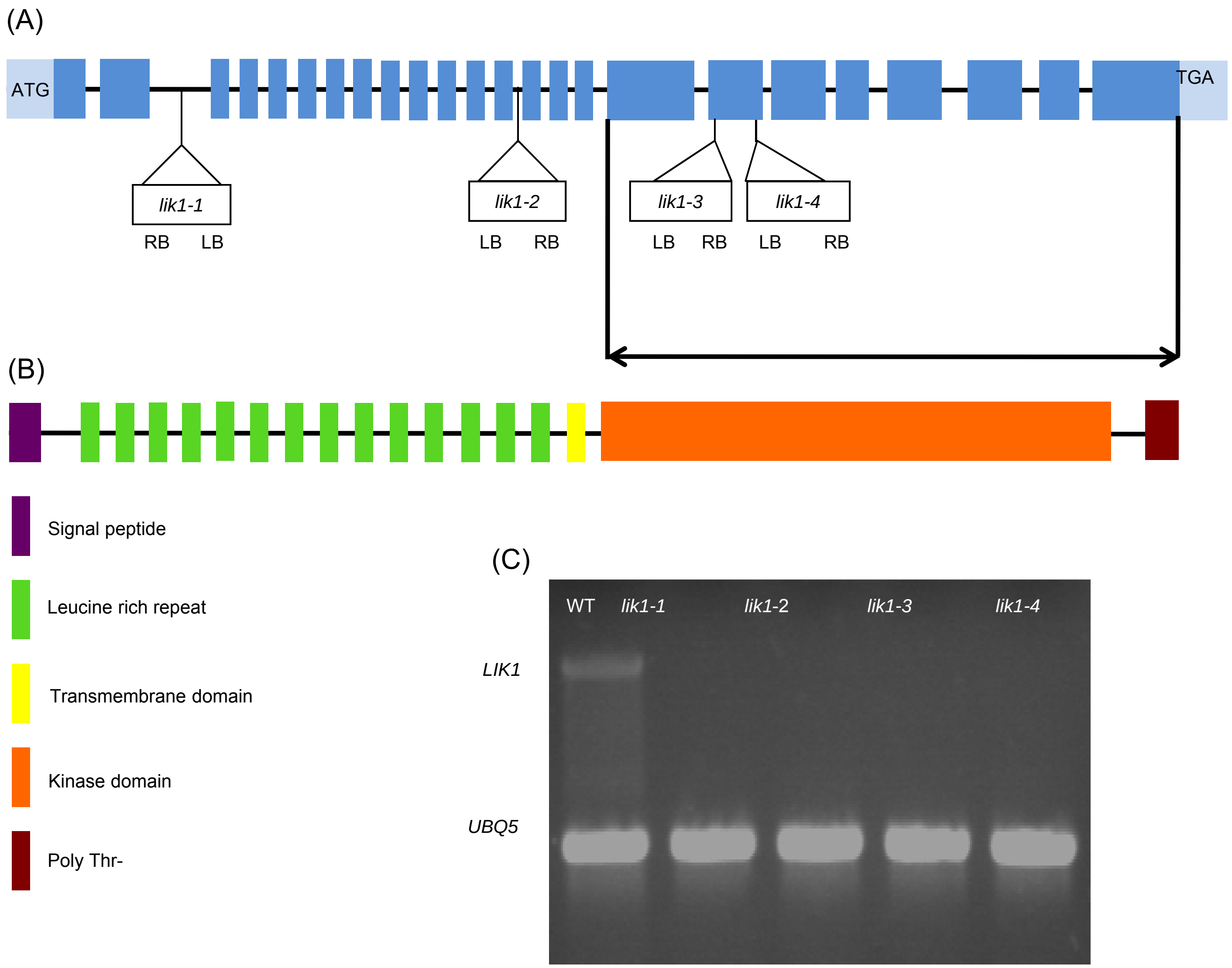

Figure S4. Analysis of *lik1* insertion mutants.

(A) A diagram of the LIK1 protein showing the locations of the various T-DNA insertion mutations (*lik1-1, lik1-2, lik1-3* and *lik1-4*).

(B) Predicted structure of the LIK1 protein, including the signal peptide, leucine-rich repeat domain, transmembrane domain, kinase domain and polythreonine tract.

(C) semi qRT-PCR analysis of the LIK1 expression in wild-type and insertion mutant from 12-day-old seedling plants. Primers were designed to amplify the kinase domain of LIK1 (as described in the Materials & Methods).
