# Supplementary figures and images for: LIK1, A CERK1-Interacting Kinase, Regulates Plant Immune Responses in Arabidopsis
Source: PLoS One. 2014 Jul 18;9(7):e102245. doi: 10.1371/journal.pone.0102245 (PMC4103824; doi:10.1371/journal.pone.0102245)

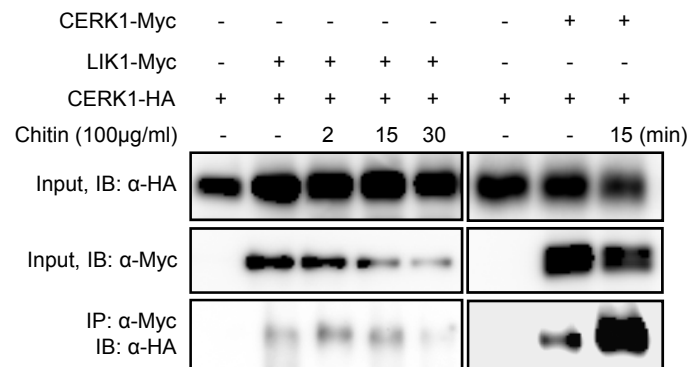

Figure S5. Second example (see also Figure 1A) of association between CERK1 and LIK1 in protoplasts.

Supplement: Figure S5 — Second example (see also Figure 1A ) of association between CERK1 and LIK1 in protoplasts. (PDF) [file pone.0102245.s005.pdf]
