# Supplementary material for: LIK1, A CERK1-Interacting Kinase, Regulates Plant Immune Responses in Arabidopsis
Source: PLoS One. 2014 Jul 18;9(7):e102245. doi: 10.1371/journal.pone.0102245 (PMC4103824; doi:10.1371/journal.pone.0102245)
Supplement: Figure S6 — Expression of LIK1 in transgenic Arabidopsis Col-0 and cerk1 mutant plants determined by western blotting. (PDF) [file pone.0102245.s006.pdf]

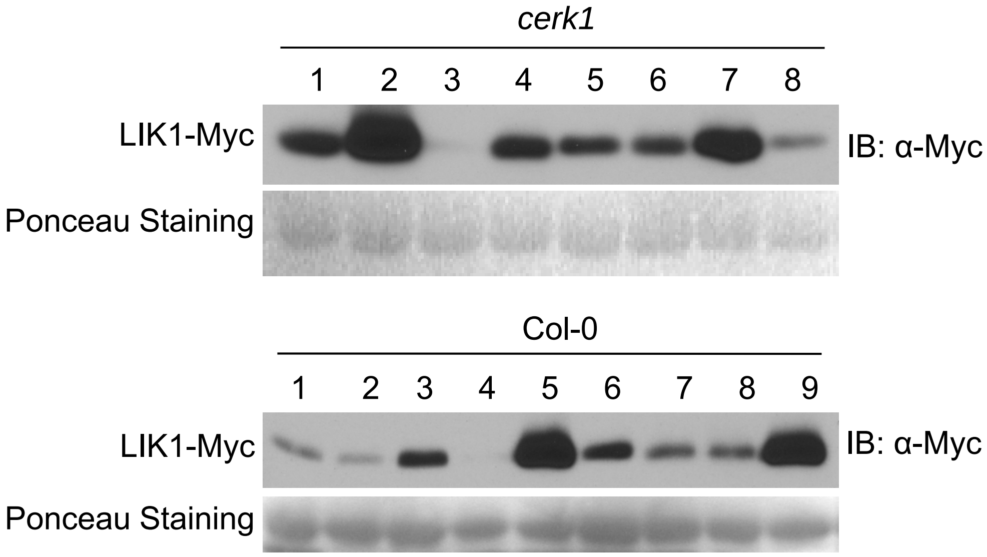

Figure S6. Expression of *LIK1* in transgenic *Arabidopsis cerk1* mutant and Col-0 wild type plants determined by western blotting.
