# Supplementary material for: LIK1, A CERK1-Interacting Kinase, Regulates Plant Immune Responses in Arabidopsis
Source: PLoS One. 2014 Jul 18;9(7):e102245. doi: 10.1371/journal.pone.0102245 (PMC4103824; doi:10.1371/journal.pone.0102245)
Supplement: Figure S7 — Lik1 mutants with enhanced ROS production in responses to chitin and flg22 elicitors. (PDF) [file pone.0102245.s007.pdf]

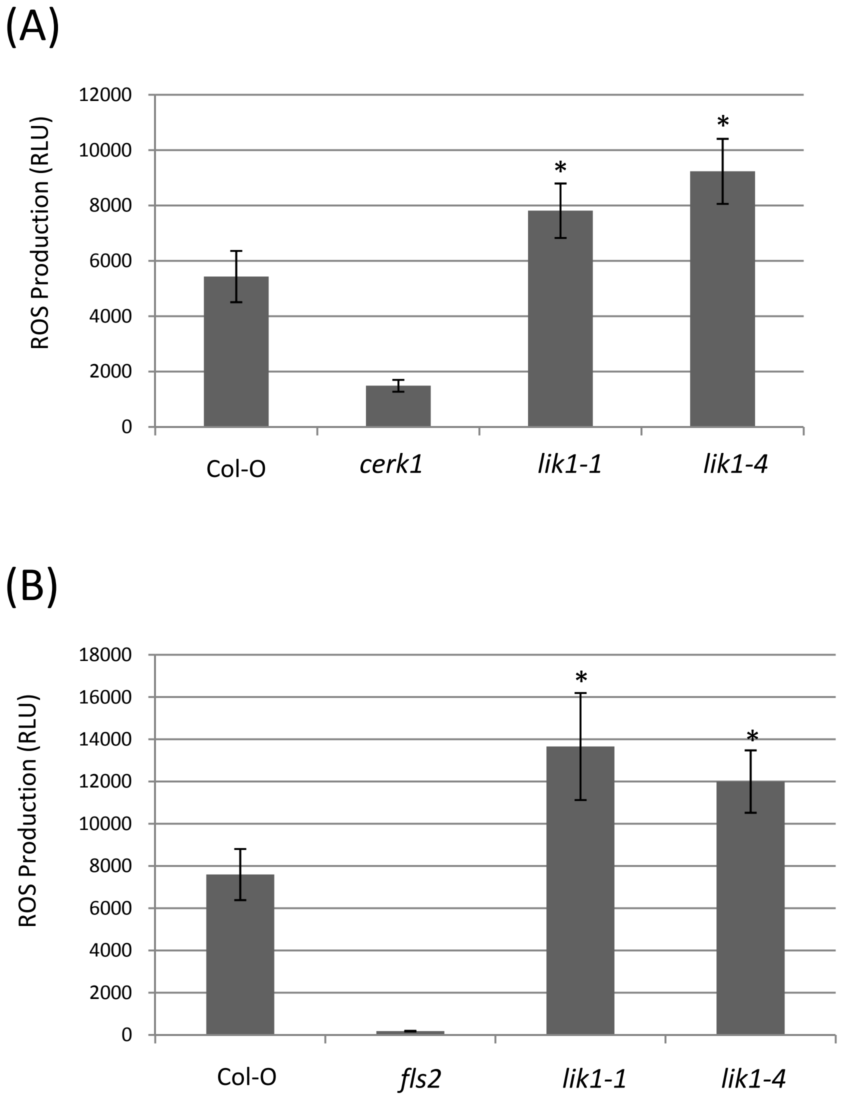

Figure S7. *Lik1* mutant plants with enhanced ROS production in responses to chitin and flg22 elicitors

ROS production in *lik1* mutant lines after treatment with chitin. ROS production was measured 20 seconds before and after the maximum signal recorded. The data are the average of the ROS signal from 32 seedlings of each mutant. These experiments were performed three times, and each replicate gave similar results. Bars represent standard deviations. Student T-test (*) P<0.05
